# MSLN Correlates With Immune Infiltration and Chemoresistance as a Prognostic Biomarker in Ovarian Cancer

**DOI:** 10.3389/fonc.2022.830570

**Published:** 2022-05-25

**Authors:** Yike Li, Wanjia Tian, Hong Zhang, Zhijian Zhang, Qinghe Zhao, Lei Chang, Ningjing Lei, Weiwei Zhang

**Affiliations:** ^1^ Department of Gynecology, First Affiliated Hospital of Zhengzhou University, Zhengzhou, China; ^2^ School of Basic Medical Sciences, Zhengzhou University, Zhengzhou, China; ^3^ Academy of Medical Sciences, Zhengzhou University, Zhengzhou, China

**Keywords:** mesothelin, ovarian cancer, prognostic biomarker, chemoresistance, bioinformatics analysis

## Abstract

Mesothelin (MSLN) is a glycoprotein with various expression degrees in different tumors including mesothelioma, ovarian cancer, pancreatic cancer, etc. MSLN is considered to play an important role in cell survival, proliferation, and tumor progression. Although the expression of MSLN in tumors makes it a potential therapeutic target, its mechanism of action is still unclear, especially its correlation with immune cells infiltration in the tumor microenvironment has not been investigated. In this study, we detected the overexpression of MSLN in ovarian cancer using database analysis and tissue-array staining. We further evaluated the diagnostic value of MSLN and found it was associated with poor overall survival in ovarian cancer. In addition, the high expression of MSLN was significantly related to the immune-related genes and chemoresistant genes. We confirmed the overexpression of MSLN in the chemoresistant ovarian cancer cell lines. Our research suggests that MSLN participates in a variety of pathways related to the suppression of immune activation and promotion of chemoresistance, leading to a poor prognosis in ovarian cancer.

## Introduction

Ovarian cancer is one of the main gynecological malignancies. According to the report of the National Cancer Institute (NCI), approximately 140,000 people die of ovarian cancer each year worldwide (1). Ovarian cancer can be divided into epithelial (90%) and non-epithelial (10-15%) types (2). According to the clinicopathological features of epithelial ovarian tumors, they can be further divided into the most common high-grade serous ovarian cancer (HGSOC, about 70%), endometrioid ovarian cancer (EOC, 10%), ovarian clear cell carcinoma (OCCC, 10%), mucinous ovarian cancer (MOC, 3%), and low-grade serous ovarian (3). HGSOC has the highest mortality rate among all ovarian cancers, showing high DNA instabilities such as TP53 and BRCA1/2 mutations (2, 4). In the clinical treatment of ovarian cancer, although chemotherapy has made a certain degree of progress, most patients with ovarian cancer are prone to recurrence and metastasis attributed to chemotherapy resistance (5). Therefore, the most effective treatment is still surgical resection in the early stage of ovarian cancer. Recent studies suggest that the tumor microenvironment composed of various immune cells and molecules is very complex, which plays an indispensable role in tumor progression and treatment. Moreover, new therapeutic applications have drawn much attention, including immunotherapy that focuses on the infiltration of immune cells in the tumor.

Mesothelin (MSLN) is a cell surface glycoprotein that was originally found to be expressed on normal mesothelial cells and mesothelioma (6). MSLN knockout mice have no abnormal developmental or reproductive issues, which suggests that this molecule is not involved in developmental regulation (7). However, the expression of MSLN is dysregulated in many types of tumors including mesothelioma, pancreatic cancer, ovarian cancer, etc. (8–11). Researchers have found that the abnormal expression of MSLN plays an important role in tumor cell growth, invasion and metastasis (12–14). For example, the high expression of MSLN in colorectal cancer can promote tumor cell proliferation (14). Studies on ovarian cancer have found that the specific binding of MSLN and carbohydrate antigen 125 (CA125) can mediate and enhance the adhesion of tumor cells, which promotes the extensive implantation and metastasis of ovarian cancer in the pelvic and abdominal cavity (13, 15). MSLN can also promote peritoneal colonization and metastasis of tumor cells by promoting tumor angiogenesis (12). In addition, MSLN has become a popular target for targeted anti-tumor therapy due to its high differential expression, including monoclonal antibodies, antibody drug combinations (ADCs), radioimmunotherapy (RIT), CAR-T cell immunotherapy for mesothelioma, etc. (16–18). Studies have shown that overexpression of MSLN can activate the PI3K pathway and induce drug resistance in pancreatic cancer cells (19). However, whether MSLN affects the drug resistance of ovarian cancer, and the related mechanisms of chemotherapy resistance are still unclear. It is also very interesting that studies on the correlation between the expression of MSLN in ovarian cancer and survival rate have opposite findings (20–22). Therefore, a systematic bioinformatics analysis of MSLN-related issues in ovarian cancer can give us more insights into the study.

In this study, we first checked the MSLN expression in 33 tumor types using the TCGA Universal Cancer Database. Since our research group is focusing on ovarian cancer studies (23), we aimed to analyze the significance of MSLN in diagnosis, clinical prognosis and chemotherapy resistance of ovarian cancer to provide novel directions for our future work. Moreover, we further found that the high expression of MSLN is significantly related to the immune-related genes and chemoresistant genes. Our studies show that as an independent prognostic factor of ovarian cancer, MSLN participates in a variety of pathways related to suppressing immune activation and promoting chemotherapy resistance, which may provide a new perspective for immunotherapy of ovarian cancer.

## Material and Methods

### Data Collection and Analysis

The clinical data of 33 tumors and 15776 tissues were obtained from the TCGA GTEx and TPM RNAseq data format from the database UCSC XENA (https://xenabrowser.net/datapages/). Through the unified processing of the Toil process (24), the RNAseq data were analyzed and compared after log2 conversion, and the Wilcoxon rank sum test was used to evaluate the differential expression of MSLN. Statistical analysis uses R software v3.6.3, and visualization uses the ggplot2 package. The cell line data GSE58470 was downloaded from the GEO database through the GEOquery package (25). One probe corresponding to multiple molecules was removed. When a probe corresponding to the same molecule was encountered, only the probe with the largest signal value was retained. We used the LIMMA R package for differential expression analyses. Genes that conformed to the |log2 (FC)| >2 and *p*<0.05 are regarded as significant differences. The top 20 or 50 differential genes were selected for visualization by heat map package (26). For all data analyses, *p* < 0.05 was considered statistically significant. (*p* ≥ 0.05, ns: no significance; *, *p* < 0.05; **, *p* < 0.01; ***, *p* < 0.001).

### Diagnostic Value Analysis

The receiver operating characteristic (ROC) curve was used to evaluate the diagnostic value of MSLN in ovarian cancer. The area under the ROC curve is between 0.5 and 1. The closer the area under the curve (AUC) is to 1, the better the diagnostic effect is. AUC has a low accuracy of 0.5-0.7, an AUC of 0.7-0.9 has certain accuracy, and an AUC of 0.9 or more has a high accuracy.

### Correlation and Enrichment Analyses

Correlation analysis was performed using the TCGA data and other mRNAs of MSLN ovarian cancer, and the Pearson correlation coefficient was calculated. The top 300 genes with the most positive and negative correlations with MSLN were selected for enrichment analysis to reflect the role of MSLN. We used the cluster Profiler package to perform the Enrich GO function for gene ontology (GO) analysis. We used the cluster Profiler package to perform the Enrich KEGG function for Kyoto Encyclopedia of Genes and Genomes (KEGG) analysis. *p* < 0.05 was considered statistically significant.

### Analysis of the Relationship Between MSLN and Immune Cell Infiltration and Immune Checkpoints

We downloaded the published data of 24 immune cell markers (27), used ssGSEA algorithm to evaluate tumor immune cell infiltration on ovarian cancer data in TCGA, and further performed Pearson correlation analysis to draw box plots and lollipop chart. We used R package **“**reshape2**”** for data processing and drew the relationship between MSLN and immune checkpoint expression and HLA molecule expression in ovarian cancer patients by R package ggplot2 and ggpubr. p < 0.05 was considered statistically significant (p ≥ 0.05, ns: no significance; *, p < 0.05; **, *p* < 0.01; ***, *p* < 0.001).

### MSLN-Related Gene or Protein Analysis

In order to understand the interaction between MSLN and other genes or proteins, we searched for MSLN in the String (https://www.string-db.org/) database to obtain the protein-protein interaction (PPI) network related to MSLN. We also used the GeneMania (https://GeneMANIA) database to analyze genes or proteins that interact or co-express with MSLN. *p* < 0.05 was considered statistically significant (*p* ≥ 0.05, ns: no significance; *, *p* < 0.05; **, *p* < 0.01; ***, *p* < 0.001).

### Evaluation of Sensitivity to Chemotherapy Drugs on Tumor

We downloaded cell line drug sensitivity data from the CellMiner database (28) (https://discover.nci.nih.gov/cellminer/) and the Genomics of Drug Sensitivity in Cancer (GDSC) (29) (https://www.cancerrxgene.org/). The database includes drug sensitivity data (IC_50_ Values). CellMiner contains 60 different human cancer cell lines, and GDSC serves as the largest public database of drug sensitivity and molecular marker information for cancer cells, with IC_50_ data for 60,434 new drugs totally. We used Pearson test to compare the relationship between the expression levels of MSLN in ovarian cancer cell lines and chemotherapy drug IC_50_ in two databases.

### Tissue Microarray Immunohistochemistry

The tissue microarray was purchased from Servicebio (China, Wuhan). This tissue microarray contains 57 cases of ovarian cancer tissues and 12 cases of normal fallopian tube tissues. The arrangement matrix and clinical information pieces of the tissue microarray are displayed in **Supplementary Table 3**. We dewaxed the tissue microarray in xylene for 20 minutes, and put the dewaxed tissue microarray in 100%, 95%, 90%, 70% ethanol, soaked each for 5 minutes, and then rinsed with double distilled water. After that, tissue antigen retrieval and inactivated enzyme treatment were performed. The goat serum was blocked at room temperature for 20 minutes, and the primary antibody for MSLN (1: 200; Abcam: ab133489), and then incubated overnight at 4°C, washed with PBS, and incubated with the secondary antibody for 30 minutes. After the DAB color development was completed, the tissue microarray was immediately rinsed to stop the reaction. Finally, the tissue microarray was sequentially subjected to hematoxylin counterstaining, dehydration, transparency, and solid sealing steps to obtain a finished product.

### Western Blot

The A2780 and A2780DDP were purchased from MEIXUAN biological science and technology (China, Shanghai). The cells were mycoplasma-free and authenticated by STR analysis. We strictly followed the instructions of the lysate kit to extract the protein, and the protein was separated on polyacrylamide gels under appropriate conditions and then transferred to polyvinylidene fluoride (PVDF) membranes (Millipore, USA). The membrane was subsequently blocked for 2 h using 5% skimmed milk and incubate the primary antibody GAPDH (1: 100000; Proteintech: 60004-1-Ig) at 4°C overnight. The next day, the diluted secondary antibody HRP-conjugated goat anti-rabbit IgG (1: 10000; Proteintech: SA00001-2) was incubated at room temperature for about 1 hour. Membranes were incubated with electrochemical luminescence (ECL) substrate (Solarbio, China) and then exposed to an X-ray film.

## Results

### MSLN Is Highly Expressed in Ovarian Cancer

**Figure 1:**
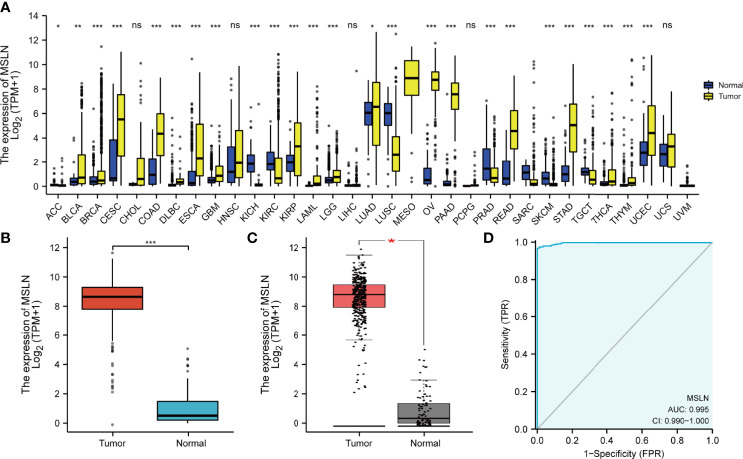
Expression of MSLN in pan-cancer. **(A)** MSLN expression in tumor and normal tissues in TCGA and GTEx pan-cancer data. **(B)** MSLN expression in OV in TCGA and GTEx pan-cancer data. **(C)** MSLN expression in OV in GEPIA2 database. **(D)** Receiver operating characteristic (ROC) curve for MSLN expression in OV. ns, *p*≥0.05; *, *p*< 0.05; **, *p*<0.01; ***, *p*<0.001, ns, no significance.

We first download the pan-cancer data of TCGA and GTEx and analyze the expression of MSLN. The analysis revealed that MSLN expression was increased in 18 tumors, including bladder urothelial carcinoma (BLCA), breast invasive carcinoma (BRCA), cervical squamous cell carcinoma and endocervical adenocarcinoma (CESC), colon adenocarcinoma (COAD), lymphoid neoplasm diffuse large B-cell Lymphoma (DLBC), esophageal carcinoma (ESCA), glioblastoma multiforme (GBM), kidney renal papillary cell carcinoma (KIRP), acute myeloid leukemia (LAML), brain lower grade glioma (LGG), lung adenocarcinoma (LUAD), ovarian serous cystadenocarcinoma (OV), pancreatic adenocarcinoma (PAAD), rectum adenocarcinoma (READ), stomach adenocarcinoma (STAD), thyroid carcinoma (THCA), thymoma (THYM), and uterine corpus endometrial carcinoma (UCEC). In contrast, its expression was downregulated in adrenocortical carcinoma (ACC), kidney chromophobe (KICH), lung squamous cell carcinoma (LUSC), prostate adenocarcinoma (PRAD), skin cutaneous melanoma (SKCM), and testicular germ cell tumor (TGCT) (**Figure 1A**). At present, MSLN is widely studied in pancreatic cancer, colorectal cancer, and other solid tumors (8–11). However, the mechanism of MSLN in ovarian cancer and immune-related bioinformatics analysis research was very limited. Our research group has focused on gynecological tumors such as ovarian cancer and cervical cancer (23, 30). Therefore, we used the R package to further analyze the TCGA database and the GEPIA2 database to confirm the high expression of MSLN in ovarian cancer (**Figures 1B, C**). Clinical characteristics related to MSLN in ovarian cancer patients are shown in **Table 1**. We further used the ROC curve to evaluate the diagnostic value of MSLN in ovarian cancer, and the results showed that MSLN had high accuracy (AUC > 0.9) in predicting ovarian cancer (**Figure 1D**; **Supplementary Table 1**).

**Table 1:** Clinical characteristics of ovarian cancer patients.

Characteristic	Low expression of MSLN	High expression of MSLN	p
**N**	189	190	
**Tumor status, n (%)**			0.006
**Tumor free**	47 (13.9%)	25 (7.4%)	
**With tumor**	122 (36.2%)	143 (42.4%)	
**OS event, n (%)**			0.010
**Alive**	86 (22.7%)	61 (16.1%)	
**Dead**	103 (27.2%)	129 (34%)	
**DSS event, n (%)**			0.004
**Alive**	91 (25.7%)	63 (17.8%)	
**Dead**	86 (24.3%)	114 (32.2%)	
**Lymphatic invasion, n (%)**			0.351
**No**	27 (18.1%)	21 (14.1%)	
**Yes**	47 (31.5%)	54 (36.2%)	
**Race, n (%)**			0.482
**Asian**	8 (2.2%)	4 (1.1%)	
**Black or African American**	12 (3.3%)	13 (3.6%)	
**White**	161 (44.1%)	167 (45.8%)	
**Age, n (%)**			0.199
**<=60**	97 (25.6%)	111 (29.3%)	
**>60**	92 (24.3%)	79 (20.8%)	
**Venous invasion, n (%)**			1.000
**No**	23 (21.9%)	18 (17.1%)	
**Yes**	35 (33.3%)	29 (27.6%)	
**Age, median (IQR)**	60 (50, 70)	58 (52, 66)	0.950

**Figure 2:**
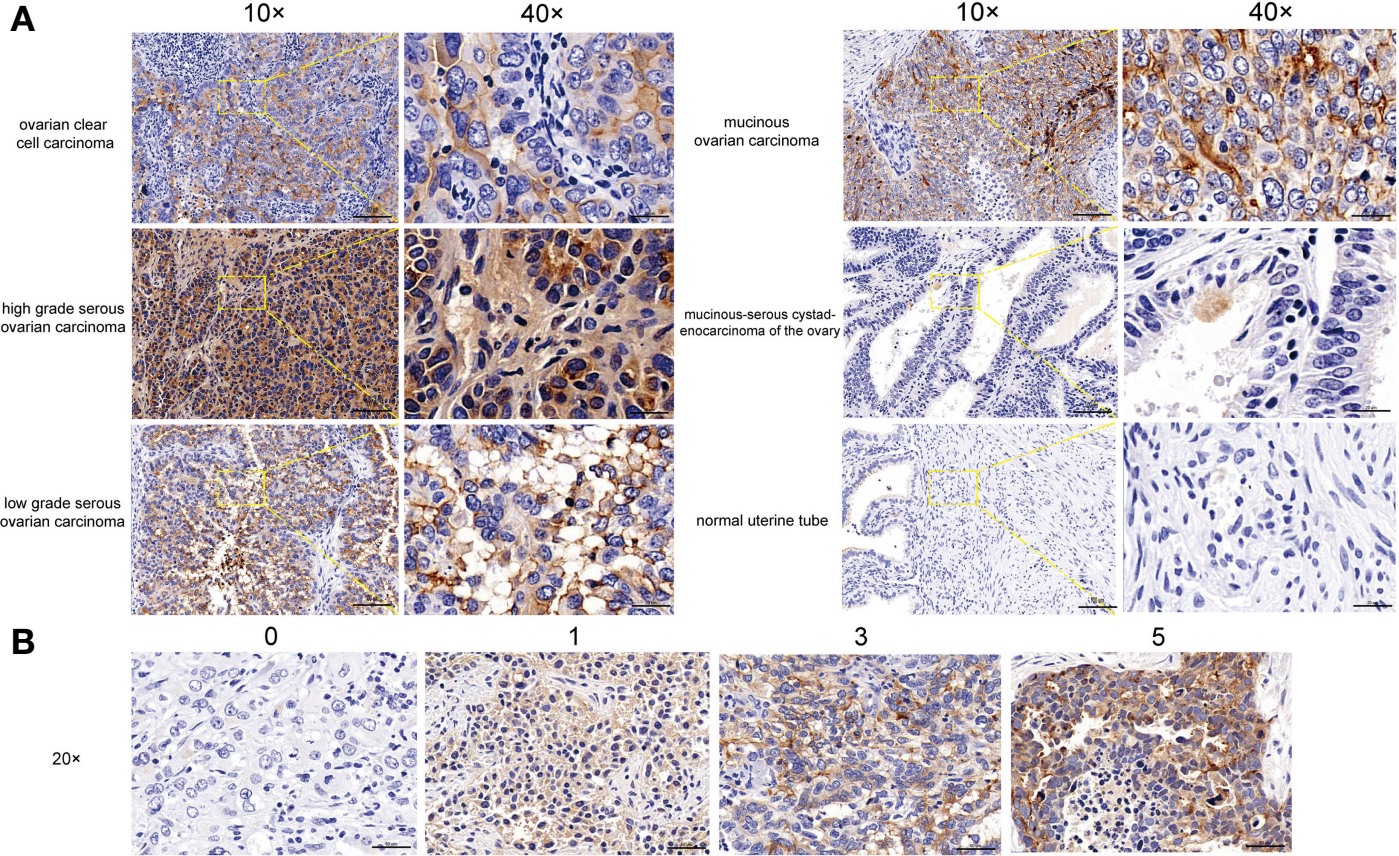
Expression of MSLN in ovarian cancer. **(A)** Expression of MSLN in tissue microarray, including ovarian clear cell carcinoma, high grade serous ovarian carcinoma, low grade serous ovarian carcinoma, mucinous ovarian carcinoma, mucinous-serous cystadenocarcinoma of the ovary and normal uterine tube. **(B)** The immunohistochemical score is scored based on the positive rate: 0 to 5% for 0 points, 6% to 25% for 1 point, 26% to 50% for 2 points, 51% to 75% for 3 points, >75% for 5 points.

We then used the tissue microarray to compare the expression levels of MSLN in different types of ovarian tissues (**Figure 2**). We found that MSLN was highly expressed in ovarian clear cell carcinoma, high-grade serous ovarian carcinoma, low-grade serous ovarian carcinoma, mucinous ovarian carcinoma, mucinous-serous cystadenocarcinoma of the ovary; in contrast, its expression is low in normal tissues **(** **Figure 2A** **)**. We scored the tissues according to the positive rate of immunohistochemistry (**Figure 2B**), and the score statistics of all sites on the tissue microarray are shown in **Table 2**. The tissue microarray arrangement matrix and clinical information are shown in **Supplementary Tables 2** and **3**.

**Table 2:** Expression of MSLN in different ovarian tissue.

Score level*	**0**	**1**	**3**	**5**
**ovarian clear cell carcinoma**	**3**	**3**	**2**	**0**
**high grade serous ovarian carcinoma**	**1**	**1**	**8**	**13**
**low grade serous ovarian carcinoma**	**1**	**2**	**7**	**7**
**mucinous ovarian carcinoma**	**2**	**4**	**1**	**0**
**mucinous-serous cystadenocarcinoma of the ovary**	**0**	**1**	**0**	**0**
**normal uterine tube**	**7**	**3**	**2**	**0**

*Score level: negative staining (0), weak staining (1), medium staining (3) and strong staining (5).

### The Correlation Between MSLN Expression and Prognosis in Ovarian Cancer Patients

**Figure 3:**
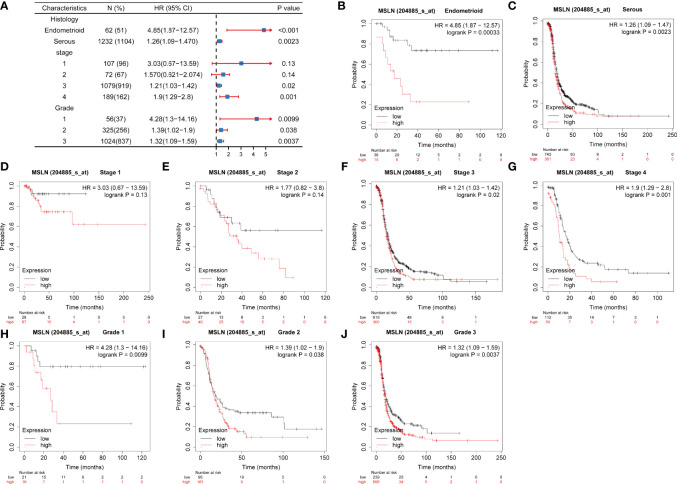
Association between MSLN expression and prognosis of ovarian cancer. **(A)** Correlations between the expression of MSLN and the overall survival rate in different stages of ovarian cancer. **(B, C)** Kaplan-Meier analysis of the relationship between MSLN and overall survival rate of ovarian cancer at different histological levels in TCGA. **(D–G)** Kaplan-Meier analysis of the relationship between MSLN and overall survival at different stages of ovarian cancer in TCGA. **(H–J)** Kaplan-Meier analysis of the relationship between MSLN and overall survival at different grades of ovarian cancer in TCGA.

In order to evaluate the value of MSLN in the prognosis of ovarian cancer patients, we analyzed the association of MSLN and the overall survival (OS) under different histologic subtype, clinical stages, and grades of ovarian cancer through an online website Kaplan-Meier Plotter. The forest map summarized the OS in different disease characteristics (**Figure 3A**; **Supplementary Table 2**). The high expression of MSLN was significantly correlated with the decrease of overall survival at the histologic subtype of in ovarian cancer (Endometrioid, p=0.00033; Serous, p=0.0023) (**Figures 3** **B, C**). The high expression of MSLN was also significantly correlated with the decrease of overall survival at different clinical stages of ovarian cancer (Stage 1, p=0.13; Stage 2, p=0.14; Stage 3, p=0.02; Stage 4, p=0.001) (**Figures 3** **D−G**). In addition, the high expression of MSLN was significantly correlated with the decreased overall survival at different grades of ovarian cancer (Grade 1, p=0.0099; Grade 2, p=0.038; Grade 3, p=0.0037) (**Figures 3** **H−J**).

### Co-Expression Gene Analysis of MSLN in Ovarian Cancer

**Figure 4:**
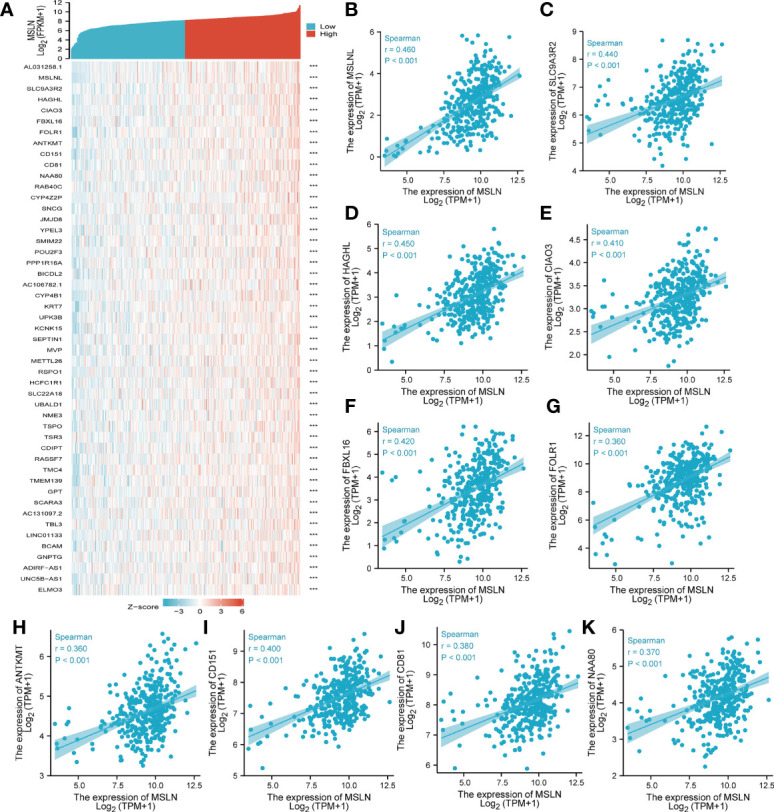
Top 50 genes positively correlated with MSLN expression in ovarian cancer. **(A)** The gene co-expression heatmap of the top 50 genes positively correlated with MSLN in ovarian cancer. **(B–K)** Correlation analysis of the top 10 genes and MALN in the heatmap. ***, *p*<0.001.

**Figure 5:**
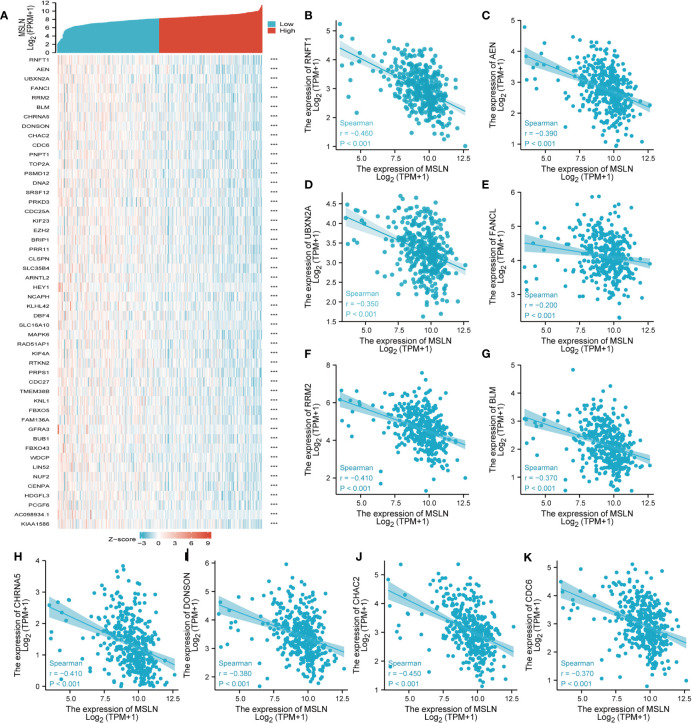
Top 50 genes negatively correlated with MSLN expression in ovarian cancer. **(A)** The gene co-expression heatmap of the top 50 genes negatively correlated with MSLN in ovarian cancer. **(B–K)** Correlation analysis of the top 10 genes and MALN in the heatmap. ***, *p*<0.001.

We explored the top 50 co-expression genes positively or negatively correlated with MSLN expression in ovarian cancer. The correlations between MSLN expression and top 10 genes expression in the heat map was further displayed and analyzed. In the heat map of positive correlation (**Figure 4A**; **Supplementary Table 4**), we obtained the top 10 genes, including MSLNL (r =0.46) (**Figure 4B**), SLC9A3R2 (r =0.44) (**Figure 4C**), HAGHL (r =0.45) (**Figure 4D**), CLAO3 (r =0.41) (**Figure 4E**), FBXL16 (r =0.42) (**Figure 4F**), FOLR1 (r =0.36) (**Figure 4G**), ANTKMT (r = 0.36) (**Figure 4H**), CD151 (r = 0.400) (**Figure 4I**), CD81 (r = 0.380) (**Figure 4J**), and NAA80 (r = 0.37) (**Figure 4K**). In the heat map of negative correlation (**Figure 5A**; **Supplementary Table 5**), we obtained the top 10 genes, including RNFT1(r =-0.460) (**Figure 5B**), AEN (r =-0.39) (**Figure 5C**), UBXN2A (r =-0.35) (**Figure 5D**), FANCL (r =-0.20) (**Figure 5E**), RRM2 (r =-0.41) (**Figure 5F**), BLM (r =-0.37) (**Figure 5G**), CHRNA5 (r =-0.41) (**Figure 5H**), DONSON (r =-0.38) (**Figure 5I**), CHAC2 (r =-0.45) (**Figure 5J**), and CDC6 (r =-0.37) (**Figure 5K**). After consulting the literature, we find that most of the genes that are positively related to MSLN are involved in cell adhesion and are related to tumor cell invasion and metastasis, such as CD81, CD151, MSLNL, SLC9A3R2, etc. And these genes are involved in related pathways such as innate immune system and class I MHC mediated antigen processing and presentation (31, 32). While most of the genes negatively related to MSLN are involved in DNA repair and cell apoptosis, involving cell cycle, mitotic, SUMOylation and other related pathways (33, 34).

### Analysis of the Functional Enrichment of MSLN in Ovarian Cancer

**Figure 6:**
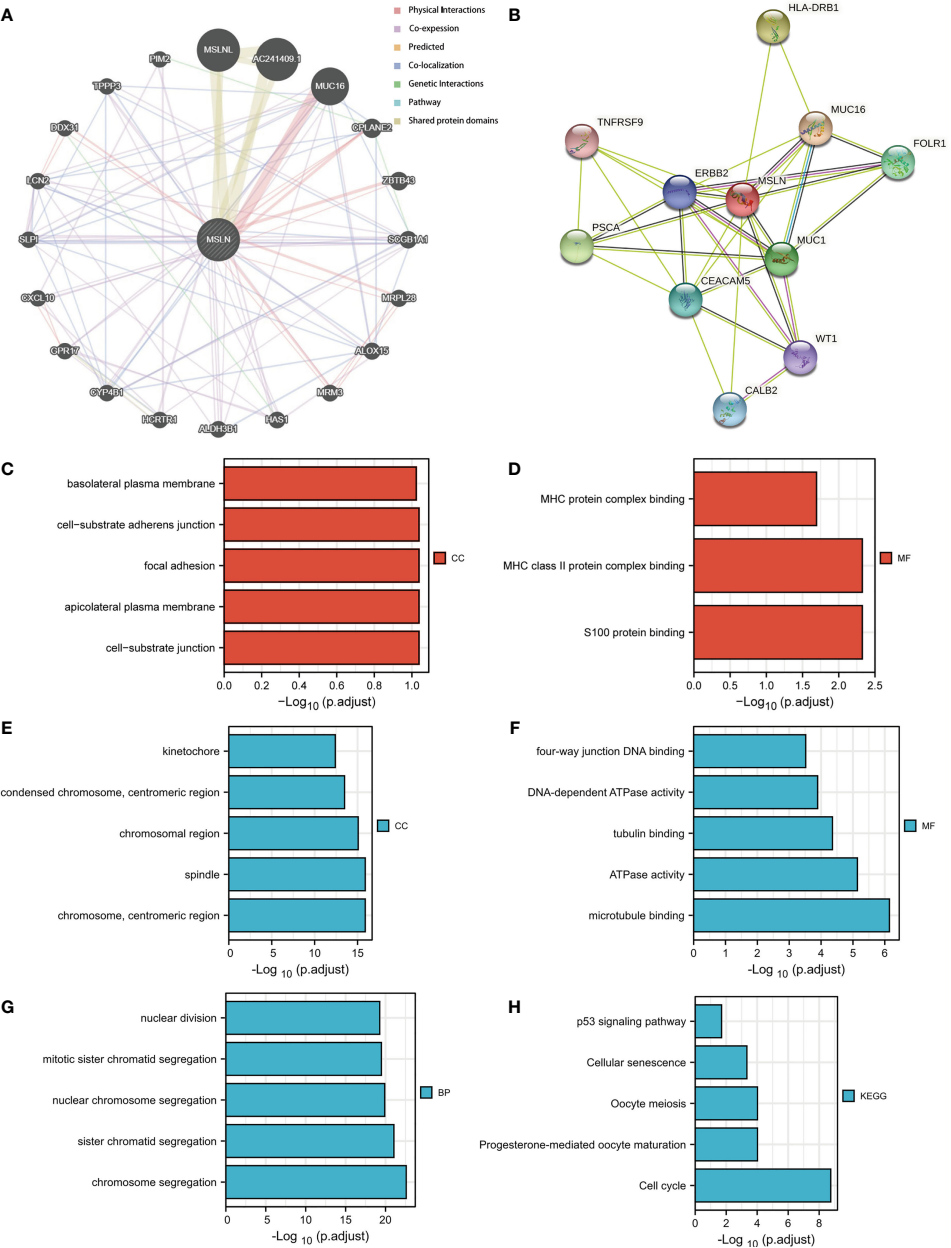
Function and pathway enrichment analyses of MSLN in ovarian cancer. **(A)**The gene-gene interaction network of MSLN was constructed using GeneMania. **(B)** A MSLN PPI network constructed using the STRING database. **(C, D)** Significant Gene Ontology terms of the top 300 genes most positively associated with MSLN, including cell component (CC), and molecular function (MF). **(E–G)** Significant Gene Ontology terms of the top 300 genes most negatively associated with MSLN, including cell component (CC), molecular function (MF), and biological processes (BP). **(H)** Significant KEGG pathways of the top 300 genes most negatively associated with MSLN.

In order to further understand the biological functions and related pathways of MSLN in ovarian cancer, we enriched and analyzed the 300 genes with the strongest positive and negative correlations of MSLN. We performed functional enrichment and PPI network analysis to clarify the interaction and co-expression mechanism between MSLN and other proteins (**Figures 6A** **, B**; **Supplementary Tables 6–8**). It was found that MSLN was co-expressed with a variety of proteins, including MUC16, HLA-DRB1, PSCA, MUC1, FOLR1, CEACAM5, CALB2, WT1, TNFRSF9, etc. The results may provide new insights for the discovery of new bio-related markers for ovarian cancer. In order to further explore the potential biological functions of the first 300 genes, we used the clusterProfiler R package to perform GO and KEGG enrichment analysis on MSLN (**Supplementary Table 9**). Cell component was mainly enriched to the basolateral plasma membrane, cell-substrate adherent junction, focal adhesion, and cell-substrate junction (**Figures 6C, E** **)**. Molecular function was primarily involved in MHC protein complex binding, MHC class II protein complex binding, S100 protein binding, and microtubule binding (**Figures 6D, F** **)**. Biological process was mainly enriched to nuclear division, chromosome segregation, and sister chromatid segregation (**Figures 6G**). The KEGG pathway enrichment was mainly related to p53 signaling pathway, cellular senescence, oocyte meiosis, and cell cycle (**Figure 6H**).

### Correlation Between Immune Cell Infiltration and MSLN in Ovarian Cancer

**Figure 7:**
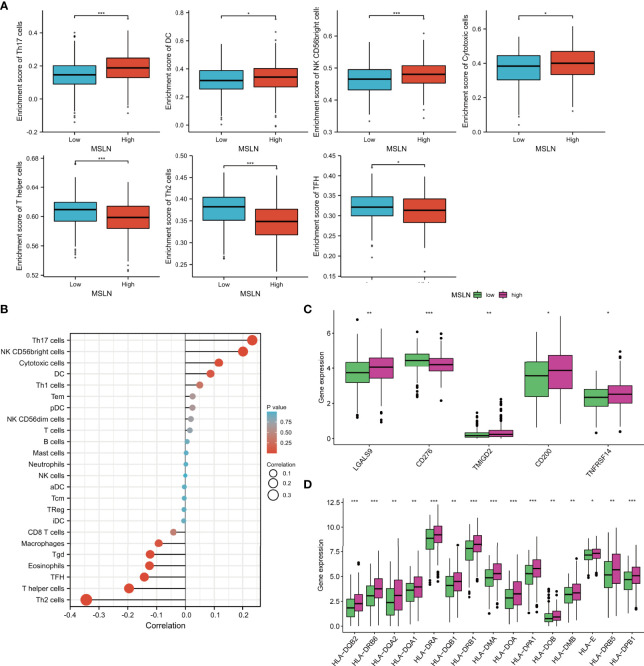
Correlation of MSLN with immune cell infiltration and immune checkpoint in ovarian cancer. **(A)** Immune cell infiltration level in the high MSLN expression group and low MSLN expression group in TCGA cohort. **(B)** Correlations between MSLN and Immune cell infiltration in ovarian cancer is shown in lollipop charts; The larger the circle, the stronger the correlation. **(C, D)** Expression level of immune checkpoint **(C)** and HLA molecule **(D)** in the high MSLN expression group and low MSLN expression group in TCGA cohort. *, *p*< 0.05; **, *p*<0.01; ***, *p*<0.001.

In order to further evaluate the relationship between MSLN and immune cell infiltration in ovarian cancer, we used the GSVA R package to perform the immune cell infiltration analysis from TCGA datasets. It was found that in the MSLN high expression group, T helper cell 17 (Th17), dendritic cell (DC), and natural killer (NK) cells had a higher degree of immune infiltration, while T helper 2 cells (Th2 cells) and follicular helper T cell (TFH) were rarely infiltrated (**Figures 7A, B**; **Supplementary Table 10**). We used TCGA ovarian cancer cohort to further analyze the relationship between MSLN and immune checkpoints. MSLN was positively correlated with immunosuppressive genes in ovarian cancer, such as LGALS9, CD276, TMIGD2, CD200, TNFRSF14 (**Figure 7C**), and human leukocyte antigen (HLA)-related families including HLA-DRA, HLA-DRB1, HLA-DPA1, HLA-DMA, HLA-E, etc. (**Figure 7D**; **Supplementary Table 11**). Studies have shown that most of these immune checkpoints play an important role in the regulation of immunosuppression and anti-tumor activity, involving related pathways including the immune regulation between the innate immune system and lymphocytes and non-lymphocytes, such as IFN-γ signaling pathway (35), IL -1 related pathways (36), Th17 cell differentiation-related pathways (37), etc. In addition, the binding of MHC class I molecules on the surface of antigen presenting cells and T cell receptor (TCR) on the surface of Th17 cells can stimulate the secretion of IL-17, TNF-α, IFN-γ and chemokines (38). Thus, macrophages, DCs and CD8^+^ T cells are recruited to promote the infiltration of these immune cells in tumor tissues to increase tumor cells apoptosis and inhibit tumor growth (37). Analysis of cytokine production in ovarian tumor cells also revealed that tumor-derived fibroblasts and antigen presenting cell secreted a variety of key cytokines to promote immune infiltration (39).

### MSLN Is Highly Expressed in Chemotherapy-Resistant Cell Lines of Ovarian Cancer

**Figure 8:**
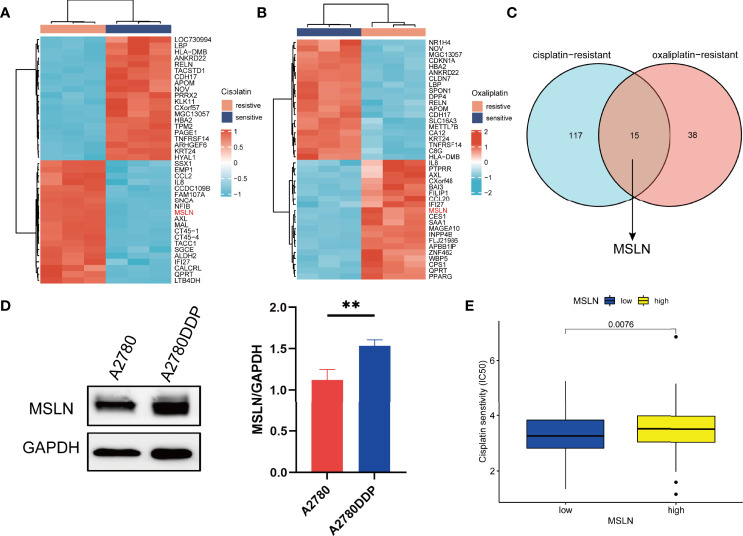
MSLN is highly expressed in chemotherapy-resistant cell lines of ovarian cancer. **(A, B)** Heat maps of the top 50 differential genes in ovarian cancer cisplatin resistance/cisplatin sensitivity and oxaliplatin resistance/oxaliplatin sensitivity based on data from GEO database. **(C)** Venn diagram of differential genes for cisplatin resistance and oxaliplatin resistance. **(D)** Western blot shows the expression of MSLN in A2780 and A2780DDP and statistical analysis of gray value. **(E)** Effect of MSLN on cisplatin sensitivity (IC50) in ovarian cancer patients was assessed by Genomics of Drug Sensitivity in Cancer (GDSC). **, *p* < 0.01.

Since chemo-resistance in ovarian cancer is a key issue in clinical practice, we also explore the correlation of MSLN and chemo-resistance. We first used GSE58470 data from GEO database to analyze the top 50 differential genes between ovarian cancer cisplatin-resistant cell lines and non-cisplatin resistant cell lines, as well as ovarian cancer oxaliplatin-resistant cell lines compared with the first 50 differential genes of normal cell lines (**Figures 8A, B**; **Supplementary Tables 14, 15**), and the Venn diagram was used to calculate the relationship between the two differential genes (**Figure 8C**). Next, we examined the expression of MSLN using a pair of ovarian cancer cell lines A2780 and A2780-DDP (cisplatin-resistant) by western blot analysis. The result showed that MSLN was overexpressed in A2780-DDP compared to A2780. (**Figure 8D**). We further evaluated the effect of MSLN on the sensitivity of cisplatin and other chemotherapeutic agents (IC50) in ovarian cancer patients by the Genomics of Drug Sensitivity in Cancer (GDSC) database. (**Figure 8E**; **Supplementary Figure 3**).

## Discussion

MSLN has been widely studied in a variety of tumors (40–43). However, MSLN in ovarian cancer and immune-related bioinformatics research is still rare. Therefore, the present study clarifies the gene expression level of MSLN in ovarian cancer, prognostic influence, related protein analysis, and immune correlation analysis.

We found that compared with normal tissues, MSLN was highly expressed in 18 kinds of tumors including ovarian cancer using a pan-cancer analysis. We then focused on the expression of MSLN in ovarian cancer by exploring the TCGA databases and GEPIA2 databases. We also detected the expression of MSLN in various ovarian tissues by immunohistochemistry staining and confirm the overexpression of MSLN in malignant ovarian cancers. We further found that the overexpression of MSLN was closely related to the poor prognosis of patients with ovarian tumors. These results indicate that MSLN is a potential biomarker for predicting the prognosis of ovarian cancer patients, which is consistent with the results of previous studies (22, 44).

In recent years, immunotherapy has opened up new horizons for tumor treatment. Although it is not clear whether tumor immunotherapy can replace traditional tumor therapy (such as chemotherapy, radiosurgery, etc.) in the short term, tumor immunotherapy does provide clinicians with new choices and new ideas (45–48). Significant progress has been made in the field of cancer immunotherapy studies and clinics in recent years, for example, Avastin has been approved by the FDA for the immunotherapy treatment of ovarian cancer (49, 50). A study also found that MSLN-directed CAR-T cell immunotherapy can provide antitumor immunity against ovarian cancer (46). Therefore, it worth further investigation about MSLN in the field of immunotherapy in ovarian cancer. Here, we found that the high expression of MSLN in ovarian cancer was positively correlated with the degree of immune infiltration of Th17 cells, DCs, and NK cells. Studies have shown that the cytokines secreted by Th17 cells can not only produce tumor-promoting effects by regulating tumor angiogenesis and improving tumor immune escape but also can play an anti-tumor effect by promoting the infiltration of CD4^+^ and CD8^+^ T cells into tumor tissues (51). Our results indicate that MSLN is closely related to immune cell infiltration, which may affect the activation of the tumor immune response that regulates the tumor progression.

Another important aspect of ovarian cancer study is to overcome the obstacle of chemoresistance. Studies have shown that knocking down MSLN in malignant pleural mesothelioma enhances the sensitivity of tumors to cisplatin (52). However, the related molecular mechanisms of MSLN in chemoresistance in ovarian cancer have not been elucidated. Here, we explore the expression of MSLN in ovarian cancer cisplatin-resistant cells, and in ovarian cancer patients with different sensitivity to various chemotherapy drugs. The information can be used for in-depth study. In recent years, poly (ADP-ribose) polymerase (PARP) inhibitor has been considered a promising anti-tumor drug (53). The combination of PARP inhibitor and chemotherapeutic agents is expected to overcome the drug resistance of tumor cells (53). A study reported that the combined use of MSLN-TTC (Targeted 227Th conjugate, radiopharmaceuticals) and PARP inhibitor may be a novel strategy for the treatment of MSLN -positive ovarian cancer (54). Thus, the functions of MSLN in ovarian cancer treatment are worth further study.

In summary, we used a large amount of TCGA database, GTEx database and GEO database information to establish the correlation and function of MSLN in ovarian cancer. We also used tissue chips composed of 57 ovarian cancer patients and 12 normal fallopian tube tissues and western blot technology to verify some of our findings. Based on our results, MSLN can be a key molecular target for new gene-targeted cancer therapy, especially in the direction of immunotherapy. It can also be a new research target for the drug resistance mechanism in ovarian cancer with further in-depth verification.

## Data Availability Statement

The datasets presented in this study can be found in online repositories. The names of the repository/repositories and accession number(s) can be found in the article/**Supplementary Material**.

## Ethics Statement

Written informed consent was obtained from the individual(s) for the publication of any potentially identifiable images or data included in this article.

## Author Contributions

This work was carried out in collaboration among all authors. NL, LC, and WZ conceptualized, designed, and directed the project. YL wrote the manuscript. YL, WT, and HZ performed the data analysis. NL, ZZ, and QZ revised the manuscript. All authors contributed to the article and approved the submitted version.

## Funding

This work was supported by Key Scientific Research Projects of Institutions in Henan Province (No.20A310019 & 20A320060), Joint Co-construction Project of Henan Medical Science and Technology Research Plan (LHGJ20190053), Henan Province Colleges and Universities Innovative Talent Support Program (No. 21HASTIT044), Henan Medical Education Project (Wjlx2020062), and Key Research and Development Project of Henan Province (No. 202102310539).

## Conflict of Interest

The authors declare that the research was conducted in the absence of any commercial or financial relationships that could be construed as a potential conflict of interest.

## Publisher’s Note

All claims expressed in this article are solely those of the authors and do not necessarily represent those of their affiliated organizations, or those of the publisher, the editors and the reviewers. Any product that may be evaluated in this article, or claim that may be made by its manufacturer, is not guaranteed or endorsed by the publisher.
