# Supplementary figures and images for: MSLN Correlates With Immune Infiltration and Chemoresistance as a Prognostic Biomarker in Ovarian Cancer
Source: Front Oncol. 2022 May 25;12:830570. doi: 10.3389/fonc.2022.830570 (PMC9174524; doi:10.3389/fonc.2022.830570)

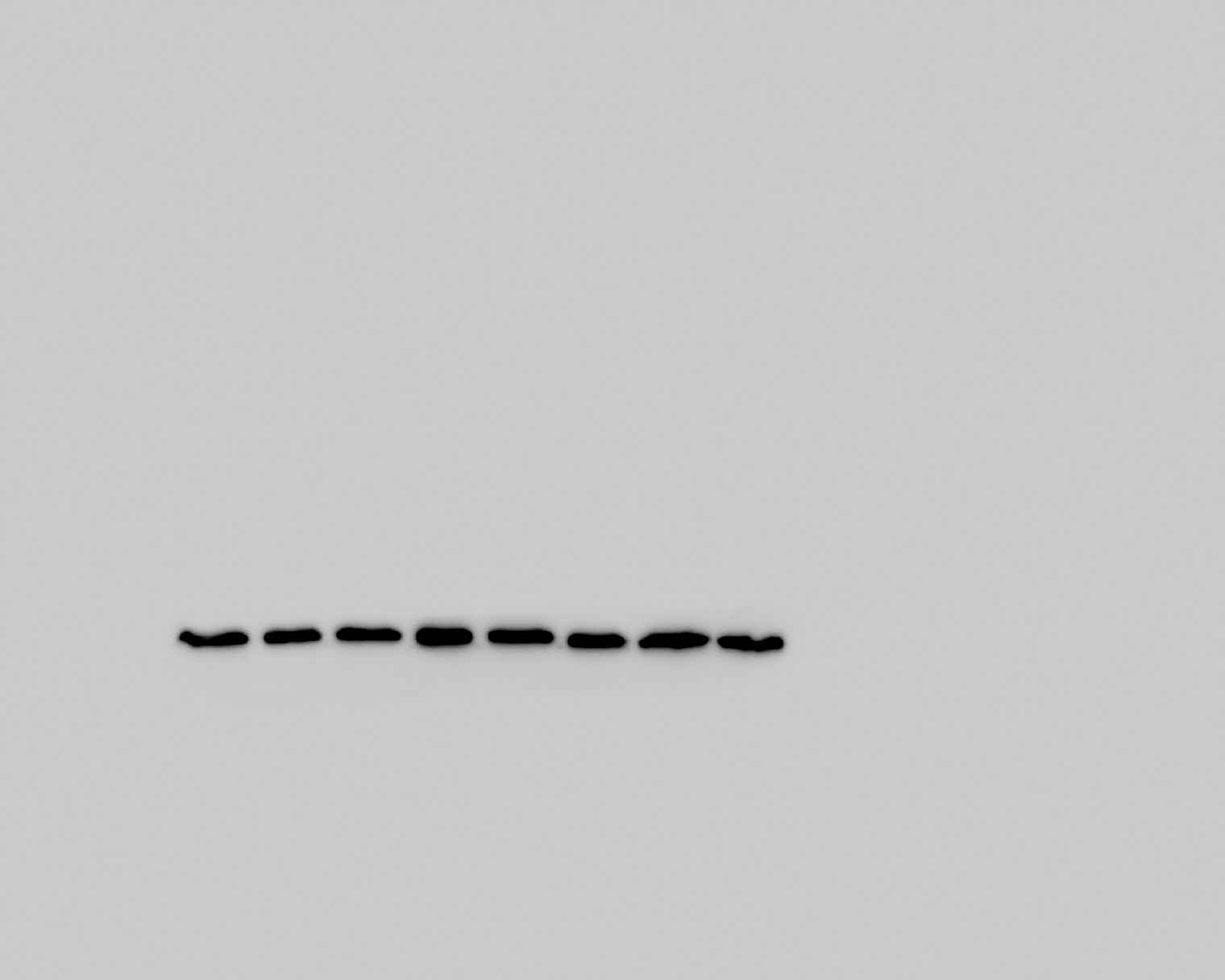

Supplement: Supplementary file 1 [file Image_1.tif]

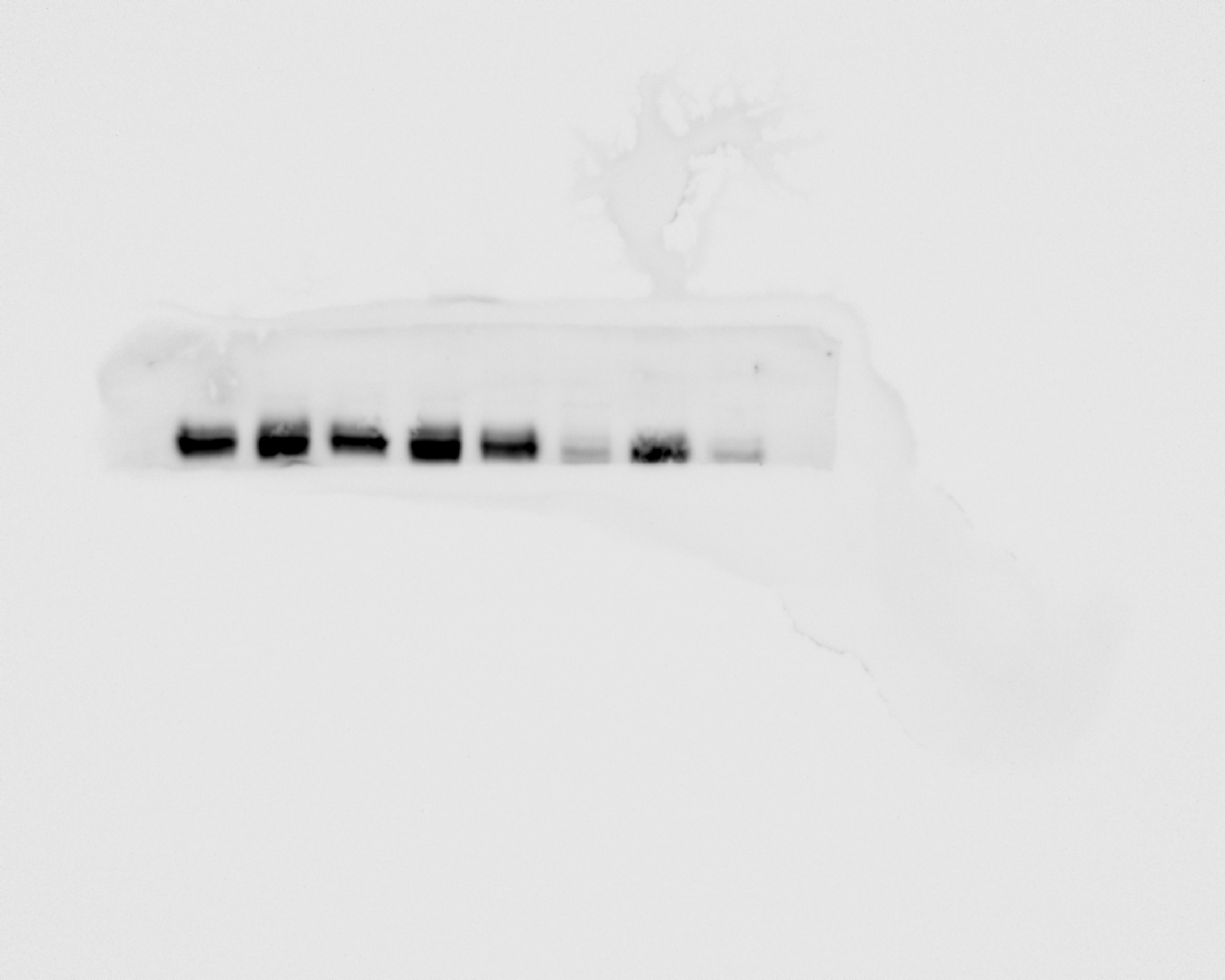

Supplement: Supplementary file 2 [file Image_2.tif]

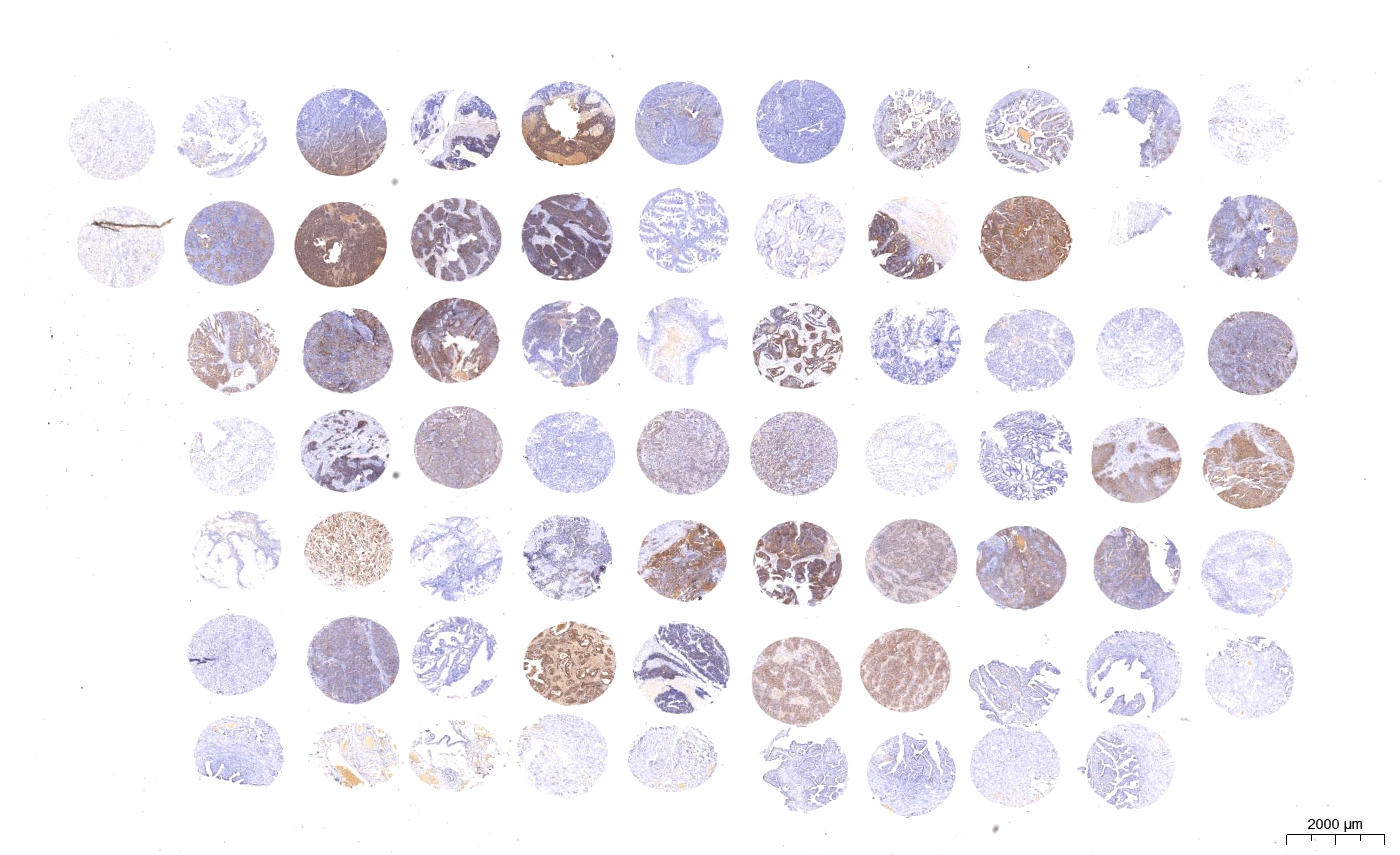

Supplement: Supplementary file 3 [file Image_3.jpeg]
